# Does the choice of EQ-5D tariff matter? A comparison of the Swedish EQ-5D-3L index score with UK, US, Germany and Denmark among type 2 diabetes patients

**DOI:** 10.1186/s12955-015-0344-z

**Published:** 2015-09-15

**Authors:** Aliasghar A. Kiadaliri, Björn Eliasson, Ulf-G Gerdtham

**Affiliations:** Clinical Epidemiology Unit, Orthopedics, Department of Clinical Sciences-Lund, Lund University, Lund, Sweden; Research Centre for Health Services Management, Institute for Futures Studies in Health, Kerman University of Medical Sciences, Kerman, Iran; Sahlgrenska University Hospital, Department of Medicine, University of Gothenburg, Gothenburg, Sweden; Health Economics Unit, Department of Clinical Sciences-Malmö, Lund University, Lund, Sweden; Department of Economics, Lund University, Lund, Sweden; Skånes universitetssjukhus, Klinikgatan 22, 22185 Lund, Sweden

## Abstract

**Objective:**

To compare the performance of the recently developed Swedish experience-based time trade-off (TTO) valuation of the EuroQol-5D-3L (EQ-5D-3L) against the hypothetical-based TTO valuations from UK, US, Germany and Denmark.

**Methods:**

Type 2 diabetes patients from the Swedish National Diabetes Register (*N* = 1 757) responded to EQ-5D-3L questionnaire in 2008. Health utilities were compared using a range of parametric and nonparametric tests. Absolute agreement and consistency were investigated using intra-class correlations coefficients (ICCs) and Bland-Altman plots. Differences in health utilities between known-groups were evaluated. Transition scores for pairs of observed EQ-5D-3L health states were calculated and compared.

**Results:**

The Swedish tariff (SWT) resulted in substantially higher health utilities and differences were more profound for more severe health problems. ICC ranged 0.6 to 0.8 and Bland-Altman plots showed wide limits of agreement. While all tariffs discriminate between known-groups, the effect sizes were generally small. The SWT had higher (lower) known-group validity for macrovascular (microvascular) complications. The SWT and UK tariff were associated with the lowest and the highest mean absolute transition scores, respectively, for 2775 observed pairs of the EQ-5D-3L health states.

**Conclusion:**

There were systematic differences between the SWT and tariffs from other countries meaning that the choice of tariff might have substantial impact on funding decisions. The Swedish experienced-based TTO valuation will give higher priority to life-extending interventions than those which improve quality of life. We suggest that economic evaluations in Sweden include both Swedish experience-based and non-Swedish hypothetical-based valuations through a sensitivity analysis.

## Introduction

Given the limited health resources, informed and transparent decision-making becomes increasingly important for health systems worldwide, and there has been a growing interest in application of economic evaluation as a tool to aid informed decision-making for health resources allocation. Cost-utility analysis is a method of economic evaluation entails assessing the effects of alternative programs or interventions on health-related quality of life (HRQoL). From a health economics perspective, a preference-based measure of HRQoL is required to estimate health-state scores and calculate quality-adjusted life years (QALYs).

Type 2 diabetes is a costly chronic disease with a growing prevalence worldwide [1]. In response to this, there has been a growing interest in evaluating HRQoL in type 2 diabetes [2, 3]. Among different disease-specific and generic measure of health outcomes, the EQ-5D-3L instrument [4] is a widely used preference-based outcome measure in the type 2 diabetes context. Recent studies suggested that the EQ-5D-3L has reasonable validity, reliability and responsiveness in type 2 diabetes [2, 5]. The EQ-5D-3L is based on five attributes: mobility, self-care, usual activities, pain/discomfort, and anxiety/depression. Each attribute has three levels: no problems, some problems, and severe problems, resulting in 243 (3^5^) possible health states [4]. Each health state is assigned an index score by applying scores from preference weights (tariffs). These tariffs can be derived from two sources: 1) individuals who actually are in the health state (experience-based valuation), and 2) a general population sample (hypothetical-based valuation).

Previous studies generally reported that the experience-based valuation resulted in higher values than hypothetical-based valuation [6, 7]. The arguments for and against using these valuation methods have been previously discussed [8–10]. Until developing the Swedish experience-based time trade-off (TTO) value set for EQ-5D-3L [11], the UK hypothetical-based TTO value set [12] has been the most commonly used in Sweden. While the choice of valuation method is a normative choice and depends on decision context, the empirical consequences of this choice are of interest as it might have critical impact on resource allocation decisions [13, 14]. To our best knowledge, only a previous study compared the newly developed Swedish tariff with the UK tariff in patients undergoing total hip replacement in Sweden and found that the Swedish tariff was valid for these patients [15].

In the current study, we used the responses to the EQ-5D-3L in a large sample of Swedish patients with type 2 diabetes to compare the Swedish experienced-based TTO valuation with hypothetical-based TTO valuations from four other countries. In our study, we answered whether the choice of TTO value sources (i.e., experienced versus hypothetical) generate any differences in health state’s utility and related health gain/loss. This is an important question since presence of any difference can have crucial impact on cost-utility analyses and funding decisions.

## Method and material

### Data

The data used in this study was collected through a cross-sectional survey conducted by the Swedish National Diabetes Register (NDR) in 2008 (IQ3 project). The Swedish version of the EQ-5D-3L was used to collect data on HRQoL among patients registered in the NDR (the visual analogue scale section of the EQ-5D-3L was not used in the survey). Twenty-six primary health care centres participated in the IQ3 project and all patients who visited one of these centres during the recruitment period (1 February to 30 May 2008) were selected to participate, as long as they met the inclusion criteria: (1) aged 18–80 years; (2) time since diagnosis longer than 6 months; and (3) not living under a protected identity. More details on the IQ3 project including sample selection have been previously presented [16]. In the current study, data on 1,757 type 2 diabetes patients who consent to participate in the study was used. Data on type 2 diabetes-related microvascular (i.e., kidney disorders and retinopathy) and macrovascular (i.e., acute myocardial infarction, heart failure, non-acute ischaemic heart disease, and stroke) complications were retrieved by data linkage with the Swedish Cause of Death and Hospital Discharge Registers. Ethical approval for the study was given by the Regional Ethical Review Board of Gothenburg, Sweden.

### Statistical analysis

The EQ-5D-3L index scores for each subject were calculated using tariffs from Sweden (SWT) [11], UK (UKT) [12], US (UST) [17], Denmark (DT) [18], and Germany (GT) [19]. The mean differences between tariffs were tested using one-way analysis of variance (ANOVA), followed by post-hoc Bonferroni tests. A minimal clinically important difference (MCID) was defined as a difference of at least 0.074 between the EQ-5D-3L tariffs based on the UKT [20]. The median and distribution of the tariffs were compared using Wilcoxon matched-pairs signed-ranks tests. Agreement between the SWT and other tariffs was evaluated using Bland-Altman plots [21] and intra-class correlation coefficients (ICCs) [22]. The ICCs were estimated using two-way random effect models. Agreement was considered poor for ICC values less than 0.40, fair for values between 0.40 and 0.59, good for values between 0.60 and 0.74, and excellent for values between 0.75 and 1.0 [23]. We also compared the tariffs in terms of ranking observed EQ-5D-3L health states using Spearman’s rank correlation.

To test for presence of any trend between severity of health state and difference between the SWT and other tariffs, the observed EQ-5D-3L health states were ranked and divided in five quintiles based on the UKT and Cuzick’s test was used to detect any significant trend [24]. We measured the absolute mean transition score in EQ-5D-3L index scores for observed pairs of EQ-5D-3L health states. This absolute transition score measures the health utility gained for a transition from a worse health state to a better health state [25]. These scores were compared using one-way ANOVA and post-hoc Bonferroni tests. In addition, we assessed the consistency of the tariffs in predicting a positive (health gain) or negative (health loss) transition scores for observed pairs of EQ-5D-3L health states. Responsiveness of the tariffs to these consistent health transitions was assessed using standardized response mean (SRM). For this, we considered a health state with lower EQ-5D-3L score as the baseline and the one with higher score as the post-treatment health states [25]. The SRM was calculated as the post-treatment mean minus the baseline mean divided by the standard deviation of the change. In addition, to assess clinical importance of the differences between transition scores by different tariffs, we computed the absolute mean differences of transition scores between SWT and other countries and compared it with the MCID of EQ-5D-3L.

Known-groups construct validity was examined by comparing the mean EQ-5D-3L index scores between groups with expected health differences. The significance of any difference was tested using t-tests. The effect size was computed as the difference between the mean of two known-groups, divided by the pooled standard deviation. An effect size of 0.2, 0.5, and 0.8 were considered as small, moderate, and large, respectively [26]. Then, we compared relative efficiency of the tariffs in discriminating between groups by calculating the ratio of the squares of these t-statistics (using the SWT as reference). Relative efficiencies lower (greater) than 1 show that the SWT had a greater (lower) ability in discriminating between known-groups. All analyses were performed in Microsoft Excel and STATA 13 (StataCorp LP, College Station, TX, USA).

## Results

The mean (SD) age of the subjects was 66.1 (8.8) years, 43 % were women, and 82 % had a BMI > 25 kg/m^2^. The prevalence of microvascular and macrovascular complications were 5 % and 24 %, respectively. The results showed that except for pain, for all other EQ-5D-3L questionnaire attributes, most patients reported no problems. 593 (34 %) subjects reported no problems on all five attributes and three (0.2 %) reported severe problems on all five attributes. The SWT and UKT resulted in the greatest and the smallest mean values, respectively (Table 1). The results of one-way ANOVA showed that the mean EQ-5D-3L index scores were significantly different across tariffs (*P* < 0.001). The mean difference (95 % CI) between the SWT and other tariffs were as follows: UKT 0.11 (0.10-0.12), GT 0.02 (0.02-0.03), UST 0.05 (0.04-0.05), and DT 0.08 (0.07-0.08). Post-hoc Bonferroni tests revealed that all these differences were statistically significant (*P* < 0.001 for the UKT, UST and DT and *P* = 0.021 for GT). The proportions of individuals with an absolute mean difference greater than the MCID in comparing the SWT with other tariffs were: 56 % with UKT, 35 % with UST, 54 % with DT, and 30 % with GT.

**Table 1:** The EQ-5D-3L index scores predicted by different tariffs

Country	Mean	95 % CI	Median	Q25	Q75	Range
Sweden	0.88	0.87-0.88	0.91	0.81	0.97	0.34-0.97
UK	0.77	0.76-0.78	0.80	0.71	1	−0.59-1
US	0.83	0.82-0.83	0.83	0.78	1	−0.11-1
Denmark	0.80	0.79-0.81	0.82	0.72	1	−0.62-1
Germany	0.86	0.85-0.87	0.89	0.79	1	−0.21-1

**Fig. 1:**
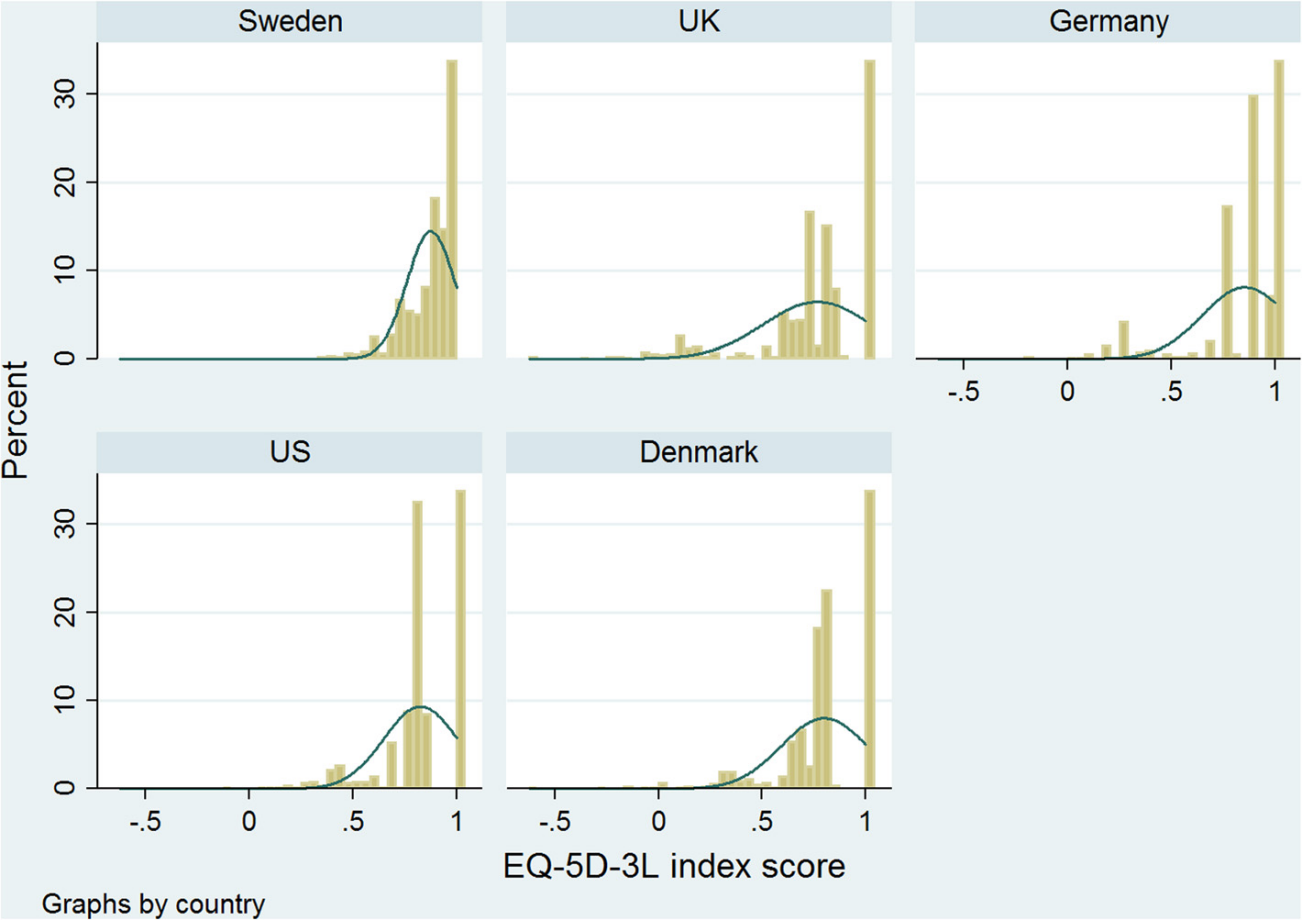
Distribution of EQ-5D-3L scores from different tariffs among type 2 diabetes patients

Figure 1 shows the distribution of the EQ-5D-3L scores using different tariffs. The SWT and GT did not show any gap between full health and the second best health state. The distribution of SWT was significantly different than the UKT, UST and DT (*P* < 0.001), but not GT (*P* = 0.16). In addition, comparing the medians these tariffs using Wilcoxon matched-pairs signed tests showed that the ST had the greatest median (*P* < 0.001).

**Fig. 2:**
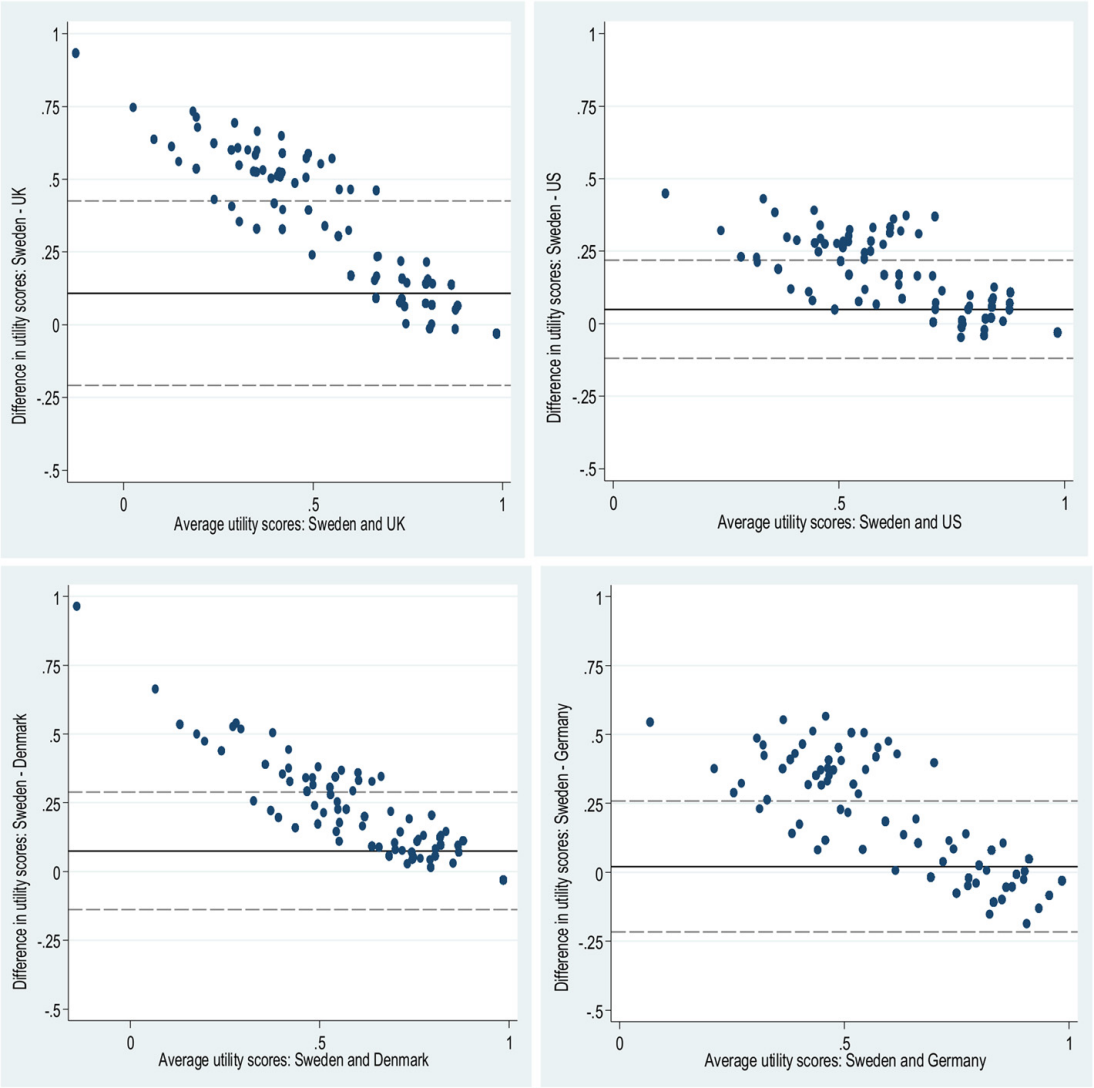
Bland-Altman plots of EQ-5D-3L scores for type 2 diabetes patients

The Bland-Altman plots (Fig. 2) showed wide limits of agreement for each pair-wise comparison. More than 92 % of the differences in individual health utility scores fell within the 95 % limits of agreement between the SWT and other tariffs. A systematic variation was observed, with a decreasing trend in the mean differences as the average EQ-5D-3L index score increased. Absolute agreement between the SWT and other tariffs was generally good with an ICC (95 % CI) of 0.60 (0.29-0.75) with UKT, 0.80 (0.62-0.88) with UST, 0.72 (0.42-0.85) with DT, and 0.74 (0.71-0.77) with GT. Although Spearman’s rank correlations between the tariffs in ranking the observed health states were high and statistically significant (*p* > 0.75, *P* < 0.001; Table 2), the correlations for the SWT were the smallest ones.

**Table 2:** Agreement between the tariffs in ranking the observed EQ-5D-3L health states measured by Spearman’s rank correlation

	Sweden	UK	US	Denmark
Sweden				
UK	0.87***			
US	0.86***	0.99***		
Denmark	0.91***	0.98***	0.97***	
Germany	0.77***	0.96***	0.96***	0.92***

***p < 0.001

In total, 75 of 243 the possible EQ-5D-3L health states were observed in our study sample. Five health states (11111, 11121, 11122, 11112, and 21121) accounted for 71 % of health states reported by the respondents. While the SWT did not result in any negative score, there was at least one health state with a negative score using other tariffs (13 UKT, 1 UST, 1 GT, and 5 DT). While the UKT and UST resulted in unique scores for every health states, there were ties (i.e., health states with the same EQ-5D-3L index scores) for other tariffs with the highest frequency for GT.

**Fig. 3:**
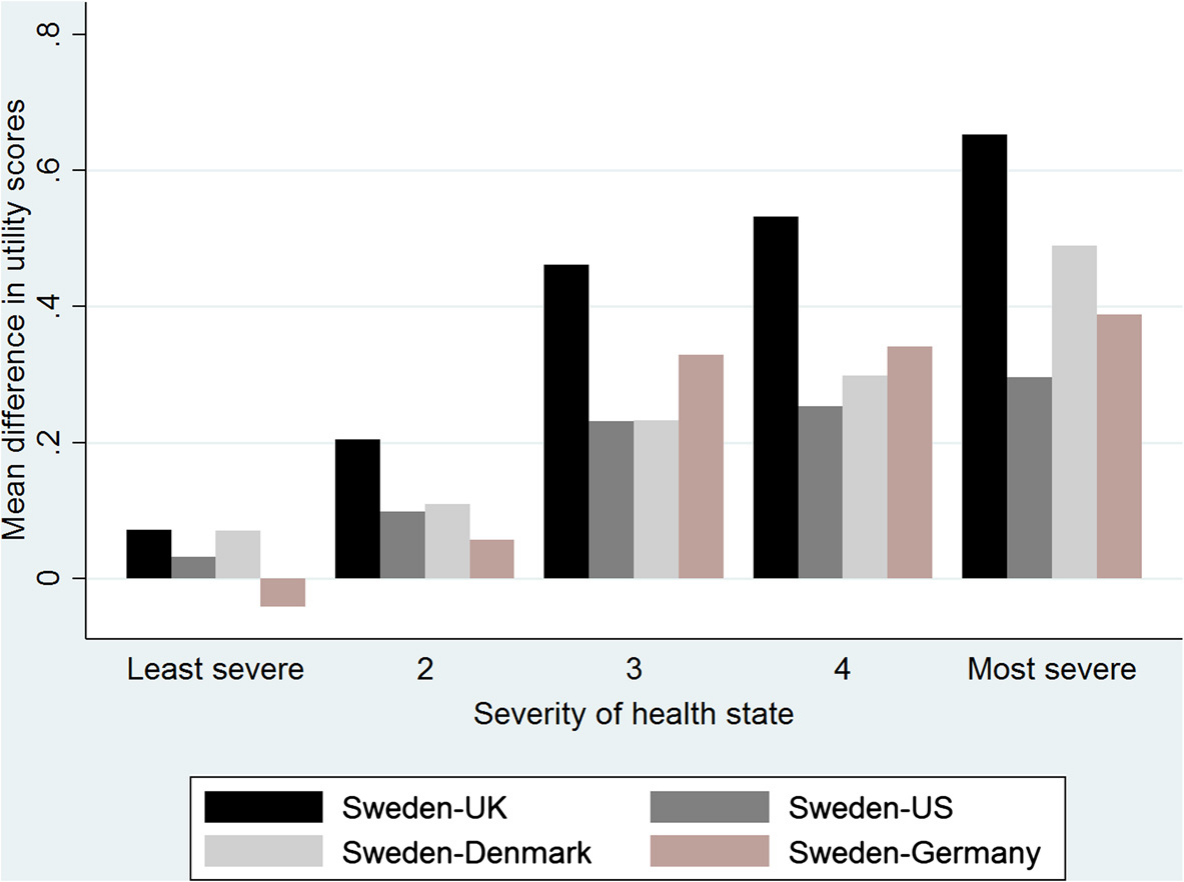
The mean difference in EQ-5D-3L scores across five quintiles of the observed health states, ranked by the UK tariff

Figure 3 displays mean difference between the SWT and other tariffs across five quintiles of observed health states ranked by the UKT. In almost all quintiles the SWT resulted in the greatest mean index scores (only in least severe health states, mean index scores for GT was greatest). There was an increasing trend between severity of health state and difference between the SWT with other tariffs and Cuzick’s test confirmed this (Z ranged 6.29 to 7.86, *P* < 0.001). Across these health states, the SWT resulted in smaller scores for 3, 6, 1, and 16 health states compared with UKT, UST, DT and GT, respectively.

The 75 observed health states resulted in 2,775 pairs of health states (_2_C_75_). One-way ANOVA and post-hoc Bonferroni tests showed that the SWT and UKT were associated with the lowest and the highest mean absolute transition scores for these 2,775 pairs (Table 3). In 2,078 of 2,775 (74.9 %) pairs of the EQ-5D-3L health states, the five tariffs were consistent in predicting health gain/loss regardless of the magnitude of the gain/loss. In addition, the SWT had the lowest and the highest consistency with GT and DT, respectively. In 1,543 of the 2,078 (74.3 %) of consistent transitions, the SWT had the smallest magnitude of gain/loss. For these consistent health transitions, SRM ranged from 1.56 to 1.81 with the lowest values for GT and SWT. A substantial proportion of differences in transition scores between SWT and other tariffs were clinically important (i.e., difference in transition scores > MCID).

**Table 3:** Absolute transition scores, consistent health transition and standardized response mean for the observed EQ-5D-3L health transitions

	Absolute mean transition score	95 % CI	Consistent health gain/loss ranked by the UK tariff (Sweden vs.) (%)	Standardized response mean for consistent transitions (*n* = 2078)	Clinically important difference in transition scores compared with Sweden^a^ (%)
Sweden	0.17	0.16-0.17	-	1.58	-
UK	0.40	0.39-0.41	84.5	1.81	76.7
US	0.26	0.25-0.27	83.4	1.73	63.7
Denmark	0.33	0.32-0.34	86.8	1.56	68.8
Germany	0.34	0.33-0.35	77.2	1.67	74.7

^a^For this, first we computed the absolute difference in transition scores between Sweden and other countries for all 2775 possible health transitions. Then, we calculated the proportion of health transitions with absolute difference greater than 0.074

Statistically significant differences (*P* < 0.05) were found for most between-group comparisons using all tariffs (Table 4). The SWT was the only one, which was not sensitive to modality of treatment. Most effect sizes were small and the relative efficiencies showed that the SWT generally had higher (lower) discriminative ability for macrovascular (microvascular) complications compared to other tariffs.

**Table 4:** Known-group validity of the EQ-5D-3L tariffs

	Sweden	UK	US	Denmark	Germany
HbA1c ≤ 7 (*n* = 890)	0.88	0.78	0.84	0.82	0.87
HbA1c > 7 (*n* = 867)	0.87	0.75	0.81	0.79	0.84
Difference	0.01*	0.04**	0.02**	0.03**	0.03**
Relative efficiency	1.00	1.29	1.25	1.37	1.25
Effect size	0.12	0.14	0.13	0.14	0.13
BMI < 30 (*n* = 991)	0.89	0.79	0.84	0.82	0.87
BMI ≥ 30 (*n* = 766)	0.86	0.74	0.80	0.78	0.83
Difference	0.03***	0.06***	0.04***	0.05***	0.04***
Relative efficiency	1.00	0.98	0.97	1.01	0.90
Effect size	0.22	0.22	0.22	0.22	0.21
Diabetes duration ≤ 8 (*n* = 929)	0.88	0.78	0.84	0.81	0.87
Diabetes duration > 8 (*n* = 828)	0.87	0.75	0.81	0.79	0.84
Difference	0.01**	0.04**	0.02**	0.03**	0.03**
Relative efficiency	1.00	1.15	1.14	1.09	1.36
Effect size	0.13	0.14	0.13	0.13	0.15
Age ≤ 67 (*n* = 929)	0.88	0.77	0.83	0.80	0.86
Age > 67 (*n* = 828)	0.87	0.76	0.82	0.80	0.85
Difference	0.01	0.01	0.01	0.01	0.01
Relative efficiency	1.00	0.41	0.81	0.41	0.84
Effect size	0.06	0.04	0.05	0.04	0.05
Microvascular complications (No) (*n* = 1670)	0.88	0.77	0.83	0.80	0.86
Microvascular complications (Yes) (*n* = 87)	0.83	0.66	0.75	0.72	0.77
Difference	0.05***	0.11***	0.08***	0.08***	0.09***
Relative efficiency	1.00	1.06	1.09	0.93	1.17
Effect size	0.42	0.43	0.44	0.40	0.45
Macrovascular complications (No) (*n* = 1332)	0.89	0.79	0.84	0.82	0.87
Macrovascular complications (Yes) (*n* = 425)	0.84	0.70	0.78	0.75	0.80
Difference	0.04***	0.08***	0.06***	0.06***	0.07***
Relative efficiency	1.00	0.76	0.78	0.69	0.85
Effect size	0.37	0.32	0.32	0.31	0.34
Myocardial infarction (No) (*n* = 1581)	0.88	0.77	0.83	0.81	0.86
Myocardial infarction (Yes) (*n* = 176)	0.85	0.71	0.79	0.76	0.81
Difference	0.03**	0.06**	0.04**	0.05**	0.05**
Relative efficiency	1.00	0.81	0.88	0.86	0.71
Effect size	0.26	0.23	0.24	0.24	0.22
Stroke (No) (*n* = 1643)	0.88	0.77	0.83	0.81	0.86
Stroke (Yes) (*n* = 114)	0.82	0.66	0.75	0.72	0.76
Difference	0.06***	0.12***	0.09***	0.09***	0.10***
Relative efficiency	1.00	0.71	0.79	0.65	0.78
Effect size	0.54	0.45	0.48	0.44	0.47
Heart failure (No) (*n* = 1673)	0.88	0.77	0.83	0.81	0.86
Heart failure (Yes) (*n* = 84)	0.82	0.65	0.74	0.71	0.75
Difference	0.06***	0.13***	0.09***	0.10***	0.11***
Relative efficiency	1.00	0.82	0.79	0.74	0.93
Effect size	0.54	0.49	0.48	0.47	0.52
Non-acute IHD (No) (*n* = 1533)	0.88	0.78	0.83	0.81	0.86
Non-acute IHD (Yes) (*n* = 224)	0.84	0.70	0.78	0.75	0.80
Difference	0.04***	0.07***	0.05***	0.06***	0.06***
Relative efficiency	1.00	0.84	0.80	0.78	0.89
Effect size	0.31	0.29	0.28	0.27	0.29
Kidney disorders (No) (*n* = 1710)	0.88	0.77	0.83	0.80	0.86
Kidney disorders (Yes) (*n* = 47)	0.82	0.61	0.72	0.69	0.73
Difference	0.06***	0.16***	0.11***	0.12	0.13***
Relative efficiency	1.00	1.28	1.33	1.13	1.43
Effect size	0.54	0.61	0.62	0.57	0.64
Retinopathy (No) (*n* = 1719)	0.88	0.77	0.83	0.80	0.86
Retinopathy (Yes) (*n* = 38)	0.84	0.69	0.77	0.74	0.80
Difference	0.04	0.08	0.06	0.06	0.06
Relative efficiency	1.00	0.85	0.98	0.79	0.82
Effect size	0.32	0.29	0.32	0.28	0.29
Insulin treatment (No) (*n* = 901)	0.88	0.78	0.84	0.81	0.87
Insulin treatment (Yes) (*n* = 856)	0.87	0.75	0.82	0.79	0.84
Difference	0.01	0.03*	0.02*	.02*	0.03**
Relative Efficiency	1.00	2.08	1.90	1.59	3.08
Effect size	0.08	0.11	0.11	0.10	0.14

*,**,*** indicates *p* < 0.05, *p* < 0.01 and *p* < 0.001, respectively

## Discussion

In the current study, we compared the Swedish experienced-based TTO valuation of EQ-5D-3L against hypothetical-based TTO valuations from the UK, US, Germany and Denmark in a large sample of Swedish type2 diabetes patients. We found wide limits of agreement between the SWT and other tariffs. The Swedish EQ-5D-3L index scores were meaningfully higher than scores produced using other tariffs. Examining the possible health transitions in our study sample revealed that the SWT, on average, would lead to smaller changes in QALYs.

The ceiling effect observed in our study is a common feature of the EQ-5D-3L questionnaire [27], implying that the EQ-5D-3L is not able to capture variations among patients in mild health states [28]. While the EQ-5D-3L was able to discriminate between known-groups, the effect sizes were small suggesting that the EQ-5D-3L might have limited discriminative ability among Swedish patients with type 2 diabetes. Moreover, there were differences between the tariffs in term of discriminative ability; and the Swedish tariff was insensitive to the modality of treatment. Lower sensitivity of experienced-based valuation was previously reported [13].

The SWT generally lead to smaller changes in QALYs, which translate into greater and less favourable incremental cost-effectiveness ratios (ICERs) compared to the other tariffs. This results from the fact that the SWT has a much narrower range than the other tariffs. There is no health state with a value close to or below zero using the SWT; the most severe health state (33333) has a utility score of 0.340. This implies that using the SWT likely result in a smaller estimates of effectiveness of interventions that substantially improve HRQoL among patients with most severe health states. In addition, higher values attached to impaired health states from the Swedish experienced-based TTO valuation result in higher utility gain from life-extending interventions and lower utility gain from quality of life-improving measures. This implies that applying the SWT in place of the hypothetical-based TTO valuations from other countries will result in more favourable ICERs for life-extending interventions and less favourable ICERs for quality of life-improving interventions [29]. Of course, there are health transitions where the SWT would be resulted in greater gains in QALYs and smaller ICERs. For example, in 270 (11.5 %) of 2345 consistent health transitions between the SWT and UKT in our study, the SWT would result in greater gain and lower ICERs. It should be noted that although we compared the Swedish experience-based tariff with other tariff in terms of differences in ICERs, an experience-based tariff may not be used to construct conventional QALYs because it does not include death as a health state [14].

Whether the different ICERs produced by the SWT compared with the other tariffs resulted in discrepant decision funding depends on several factors including efficacies of interventions, the severity of the health states, the distribution of health transitions, cost difference between intervention and the willingness to pay (WTP) threshold. For example if we assume a WTP of Swedish Krona (SEK) 500,000 per QALY gained, then if a new intervention is SEK 1000 more expensive and causes the health transitions from the health state “23231” to the health state “23222”, then the ICER applying the SWT and UKT will be SEK 1,428,571 and 5208 per QALY gained, respectively, that translate into different decision funding. However, in this example, if the WTP changes to SEK 1,500,000, or cost difference decline to SEK 100, or the health transition happens in a minor portion of the sample, then difference in QALY gained between the SWT and UKT does not result in discrepant decision funding.

The narrower range of the SWT compared with the other tariffs might be due to the difference in valuation method (experienced vs. hypothetical). Previous studies found that patients generally report higher values than general population and the difference increased as health states gets worse [7, 30, 31]. The similar trend was observed in the present study. The differences between valuation elicited from patient and general population are attributed to failure to rate the same health state, to have different measuring rods, and patient adaptation to a health state [10]. The differences in methodology including data collection and estimation models might be another reason for the observed difference between the SWT and the other tariffs here. For example, the data for the SWT was collected by self-administered postal survey while in other countries interviews were used. Moreover, due to methodological limitations in the surveys, the SWT did not allow for negative values which should be taking into account in any application. Differences induced by translation and inherent difference in populations (e.g., cultural norms, socioeconomic status, health status, clinical practice patterns, access to health care services) might be other potential explanations of observed difference comparing the SWT with the other tariffs [32].

The results of the current study should be interpreted in light of some limitations. The study was conducted among people with type 2 diabetes and only 75 (31 %) of 243 the possible EQ-5D-3L health states were observed. While our sample demographic and clinical characteristics are comparable with the Swedish type 2 diabetes population (www.ndr.nu), but possible differences in other characteristics (e.g., socioeconomic status) might limits generalizability of our findings to all Swedish type 2 diabetes population. In addition, our finding might not be generalizable to other diabetes population or other diseases where the other EQ-5D-3L health states might be more common. As a consequence of cross-sectional design of the study and lack of data on changes in health status over time, we assumed that all health transitions had the same probability of occurrence that is not true in reality. In addition, we were not able to assess the responsiveness to change and test-retest reliability of the tariffs.

## Conclusion

We found that there were substantial and clinically important differences between the Swedish experienced-based TTO valuation of EQ-5D-3L and the hypothetical-based TTO valuations from other countries and the differences were larger for more severe health states. This finding and also wide limits of agreement between the tariffs suggest that choice of tariff might have important impact on economic evaluation studies and funding decisions. We did not aim to answer the question of which valuation (experienced or hypothetical) should be used to health state for cost utility analyses. However, if based on recommendation by the Swedish Dental and Pharmaceutical Benefits Agency, the Swedish experienced-based TTO valuation is used in cost-utility analyses, we suggest that the hypothetical-based TTO valuation should be applied in a sensitivity analysis and the impact on the finding should be discussed. Further analyses are needed to explore the factors contributed in the observed differences in the present study. Comparing different tariffs in a longitudinal study, the influence of differences between tariffs on health studies including economic evaluation and inequality, and differences in EQ-5D-5L tariffs whenever the Swedish EQ-5D-5L value set become available are subjects for future studies.
